# Preparation of Magnesium-Aluminum Hydrotalcite by Mechanochemical Method and Its Application as Heat Stabilizer in poly(vinyl chloride)

**DOI:** 10.3390/ma13225223

**Published:** 2020-11-19

**Authors:** Yinan Jiang, Zhanhong Yang, Qingsong Su, Linlin Chen, Jian Wu, Jinlei Meng

**Affiliations:** 1 Institute of Chemical Power and Materials, College of Chemistry and Chemical Engineering, Central South University, Changsha 410083, China; jiangyinan17@csu.edu.cn (Y.J.); qingsongsu@outlook.com (Q.S.); chenlinlin4321@163.com (L.C.); 192301027@csu.edu.cn (J.W.); csumengjinlei@163.com (J.M.); 2 Institute of Chemical Power and Materials, Innovation Base of Energy and Chemical Materials for Graduate Students Training, Central South University, Changsha 410083, China

**Keywords:** poly(vinyl chloride), magnesium-aluminum layered double hydroxide, carbon dioxide, mechanochemical method, thermal stability

## Abstract

The traditional methods for preparing magnesium aluminum layered double hydrotalcite (Mg_2_Al-CO_3_LDHs) in industry include coprecipitation and hydrothermal methods. Both these methods have the disadvantages of high preparation cost and complicated water washing process. Using Mg(OH)_2_, Al(OH)_3_, and CO_2_ as raw materials in this work, the Mg_2_Al-CO_3_ LDHs are successfully prepared by mechanochemical method, which solves the shortcomings of traditional preparation method and realizes the conversion and utilization of CO_2_ resource. The prepared Mg_2_Al-CO_3_ LDHs are evaluated as a heat stabilizer in poly(vinyl chloride) (PVC). The result indicates that, when 2.4 phr Mg_2_Al-CO_3_ LDHs, 0.3 phr ZnSt_2_, and 0.3 phr of zinc acetylacetonate are added to the PVC, the thermal stability time of PVC can reach 190 min, which is better than PVC containing commercial Mg_2_Al-CO_3_ LDHs. Meanwhile, its processing performance is basically the same as the PVC containing commercial Mg_2_Al-CO_3_ LDHs.

## 1. Introduction

Heat stabilizer is one of the most important categories in plastic additives, which is in sync with the birth and development of poly(vinyl chloride) (PVC). There are many types of heat stabilizers, among which traditional heat stabilizers include organic tin, organic antimony, lead salts, metal soaps, etc. [1,2]. Organotin-based heat stabilizers have excellent thermal stability, weather resistance, initial colorability, nontoxicity, transparency, among other excellent properties, and are currently the most widely used, most effective and promising class of heat stabilizers. However, its expensive price limits its widespread application [3,4]. Organic antimony stabilizers have good thermal stability, low price, and low toxicity, but they have poor light stability, and lubricity [5]. Lead salt stabilizers were widely used in PVC products due to their low cost and excellent performance. However, lead is a heavy metal that is harmful to humans and the environment [6]. Metal soap heat stabilizers are mainly Ca-Zn composite heat stabilizers. However, they may also have a “zinc burn” phenomenon even though they are inexpensive and non-toxic [7]. So, they need to be formulated with another auxiliary heat stabilizer to make a multi component complex formulation when added to PVC [8]. In general, there is an urgent need for a material that is not only inexpensive, non-toxic, and environmentally friendly, but also has excellent overall performance.

Layered double hydroxides (LDHs), also known as hydrotalcite-like compounds, are a class of compounds characterized by a layered structure. It has the generic formula ${\left[M_{1-x}^{2+}M_{x}^{3+}{\left(\mathrm{OH}\right)}_{2}\right]}^{x+}{\left(A^{n-}\right)}_{x/n}\cdot {\mathrm{mH}}_{2}O$ where x = 0.2~0.33, M^2+^ is a divalent metal cation, M^3+^ is trivalent cation, and A^n−^ is non-framework charge compensating anion. Particularly, its structure may be positively charged by substituting a portion of divalent cations in the brucite lattice with trivalent cations, which the intercalation of anions between the layers balance the charge [9,10]. Due to its special layered structure, LDHs have controllability in the chemical composition of the cationic sheets, the type and number of the intercalated anions, and the size and distribution of the grains. Therefore, there are many kinds of hydrotalcites, which have broad application prospects in the fields of adsorption [11], medicine [12], catalysis [13], electrochemistry [14,15,16], flame-retardant [17,18], and photochemistry [19]. Moreover, it can also be used as a heat stabilizer for PVC [20]. In the 1980s, hydrotalcite was firstly found that when added to PVC materials had better thermal stability than PVC materials without adding hydrotalcite [10]. This set off a research upsurge concerning the application of hydrotalcite in PVC resin. Nowadays, as a typical layered double hydroxides, Mg-Al-CO_3_-LDHs has been widely used as a high-efficiency long-term stabilizer for calcium and zinc soap heat stabilization systems [21]. Generally, the synthesis methods of Mg-Al-hydrotalcite-based heat stabilizers include the co-precipitation method [22], ion exchange method [23], urea method [10], hydrothermal method [24], roasting recovery method [25], and nucleation/crystallization separation method [26]. However, these synthetic methods require a large amount of salt solution and alkaline solution, which results in the need to wash with a large amount of water in the later stage and produce low-value salt solutions and alkaline solutions. The production cost is too high and the raw material utilization rate is low. This runs counter to the goal of reducing production costs and environmental friendly.

Nowadays, the world-population and energy consumption are continuous increasing, which will consume a large amount of fossil energy and release the greenhouse gas CO_2_, causing serious environmental problems such as global warming. For the sustainable development of human society, it is urgent to develop new green technologies to realize the conversion and utilization of CO_2_ resource [27].

In this work, traditional corresponding salts were substituted with Mg(OH)_2_ and Al(OH)_3_ and CO_2_ was used as carbon sources, Mg_2_Al-CO_3_ LDHs were prepared by mechanochemical methods, the possible synthesis mechanisms were discussed as well. Meanwhile, the prepared Mg_2_Al-CO_3_ LDHs were formulated with Zn-based soap initial heat stabilizers to make multicomponent complex formulation and added to PVC to evaluate the thermal stability performance.

## 2. Experimental

### 2.1. Materials

PVC resin (S-65) was industrial grade (Formosa Plastics Co. Ltd., Ningbo, China). Mg(OH)_2_, Al(OH)_3_ and CO_2_ were of A.R. grade and dioctyl-phthalate (DOP). The water used in the experiment was deionized water.

### 2.2. Preparation of Hydrotalcite

Briefly, 11.6g (0.2 mol) Mg(OH)_2_ and 7.8 g (0.1 mol) Al(OH)_3_ were mixed, the grinding ball and the mixture were put into the grinding jar according to the ball-to-powder weight ratio of 10:1. The rotation speed was set at 400 r/min. After 5 h of ball milling in a planetary ball mill, the solid powder was transferred into a three-necked flask, then 100 mL water was added, 2.2 g (0.05 mol) CO_2_ was passed into the solution, the pH was controlled at 11. After stirring in a 95 °C water bath for 30 h, the reactant was filtered, washed, dried in an 80 °C drying oven for 12 h, and ground for use.

### 2.3. Material Characterization

The X-ray diffraction (XRD) analysis was performed using a D8 ADVANCE diffractometer (Bruker, German) with Cu-K_α_ targets (λ = 1.5406 Å) at a scanning rate of 8°/min, with 10~80° of the scanning range, and operated at a voltage of 40 kV and current of 40 mA.

The surface morphology of the samples were investigated via scanning electron microscope (SEM) study. The SEM measurements were carried out in Mira3 scanning electron microscope (Tescan, China). All samples were coated with a thin layer of gold prior to testing.

The fourier transform infrared spectroscopy (FTIR) study of the samples were carried out using a Nicolet iS5 (Thermo Fisher, Waltham, MA, USA) spectrometer at room temperature. The samples were crushed well and then examined in KBr pellets.

A TGA5500 thermogravimetric analysis instrument (Wakefield, MA, USA) was employed to analysis the sample stability, the sample was heated under an air flow from room temperature to 600 °C at a constant rate of 5 °C min^−1^.

The sample size was analyzed with MS2000 laser particle size analyzer (Malvern, UK). The sample was dispersed in absolute ethanol. After ultrasonication for 1 h, it was placed in the sample cell for particle size testing.

### 2.4. Thermal Stability Testing of PVC–LDHs Composites

The static thermal aging test, Congo red test and thermal weight loss test were used to evaluate the thermal stability of PVC-LDHs composites. The specific steps of the static thermal aging test were as follow: 100 g PVC resin, 70 mL DOP and a certain of stabilizers were mixed evenly and then added in a SK-160B plastics mixing mill (Shanghai Light Industry Machinery Co. Ltd., Shanghai, China) for 5 min at 175 °C to form films. In addition, a group of sample without heat stabilizers was as comparison. After that, the films were cut into 2 × 2 cm^2^ strips, which could be placed into a 180 °C oven for static thermal aging test. The color changes of the composites were recorded by a digital camera every 10 min. According to ISO standard 182-1:1990 (E), the specific steps of Congo red test were as follow: 2 g PVC strips were cut into pieces and added into a test tube, which a Congo red test paper was located at 3 cm above the sample. Then, the test tube was immersed in an oil bath at 190 °C to evaluate the thermal stability of the sample. The time required for the Congo red paper to change from red to blue was recorded and repeated three times to take the average. The specific steps of the thermal weight loss test were as follows: 10 g PVC resin, 7 mL DOP, and certain stabilizers were mixed evenly and then transferred into the porcelain boats. The porcelain boats were placed into an oven with a temperature of 190 °C and took out every certain time to weigh and calculate the weight loss.

## 3. Results and Discussion

### 3.1. Characterization of Mg_2_Al-CO_3_ LDHs

**Figure 1:**
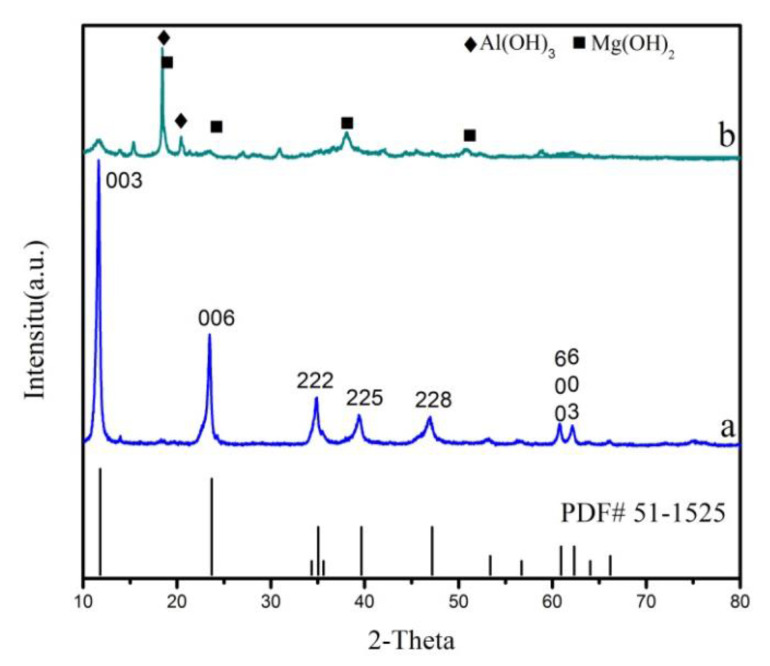
XRD patterns of Mg_2_Al-CO_3_ LDHs prepared with (**a**) CO_2_ and (**b**) noneas carbon sources.

In order to explore the crystal form of the prepared sample and the mixture after ball milling, XRD analyses of the samples are carried out and the result is shown in Figure 1. It can be clearly seen from the figure that the sample a has obvious characteristic diffraction peaks at 2θ = 11.66°, 23.46°, 34.80°, 39.38°, 46.84°, 60.72°, and 62.13, respectively, corresponding to the 003, 006, 222, 225, 228, 600 and 603 crystal planes, which matches the standard card (Chao and Gault, 1997) of Mg_4_Al_2_(OH)_12_CO_3_·3H_2_O (PDF# 51-1525). According to Bragg’s law, the d-values between standard card and prepared LDHs are indicated in Table 1. As shown in Table 1, the d-values of the standard card and the prepared LDH at the 003, 006 crystal plane are almost identical, which indicates that the sample a is typical hydrotalcite layered structure. The baseline is stable, the peak width is narrow and sharp, and there is no obvious impurity peak, indicating that the Mg_2_Al-CO_3_ LDHs are successfully synthesized. Curve b has strong Mg(OH)_2_ and Al(OH)_3_ characteristic diffraction peaks, and there is a weak and wide diffraction peak at 2θ = 11.66°, corresponding to the 003 crystal planes of Mg_4_Al_2_(OH)_12_CO_3_·3H_2_O. It indicates that there is almost no reaction of the raw materials in the sample, and only a small amount of amorphous LDH is generated. The reason for this is that, under mechanical force effects, such as impact force, shear force, pressure, etc., the mixture sample will undergo crystal lattice distortion and particle amorphization, and its structure will form an amorphous layer after being strongly damaged.

**Figure 2:**
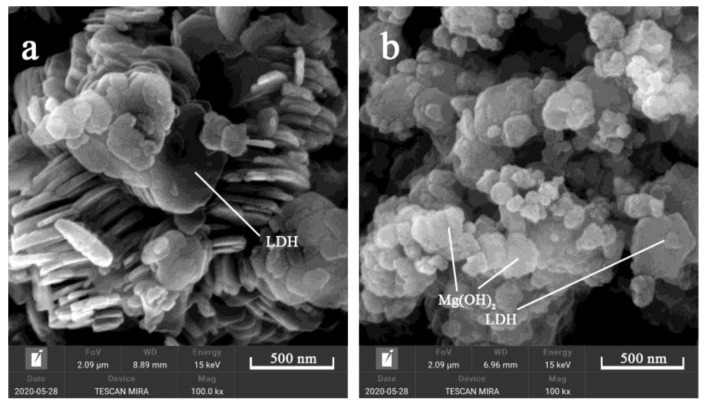
SEM images of Mg_2_Al-CO_3_ LDHs prepared with (**a**) CO_2_ and (**b**) noneascarbon sources.

In order to explore the morphology of the samples, SEM microphotographs of the samples are shown in Figure 2. It can be clearly seen from the images that the morphology of sample a exhibits a regular hexagonal structure, and the sheets are stacked together, which is a typical layered hydrotalcite structure. The crystal size of sample a is 300~500 nm and the thickness is about 30 nm. It shows that sample a has small crystal size, and its size is uniform, the crystal morphology is regular, which is consistent with the XRD pattern result. Sample b exhibits a flaky structure with only a small amount of hexagonal structure and uneven crystal size, indicating that mechanical grinding cannot directly synthesize Mg_2_Al-CO_3_ LDHs, and it needs to be crystallized for a certain time under certain temperature conditions.

**Figure 3:**
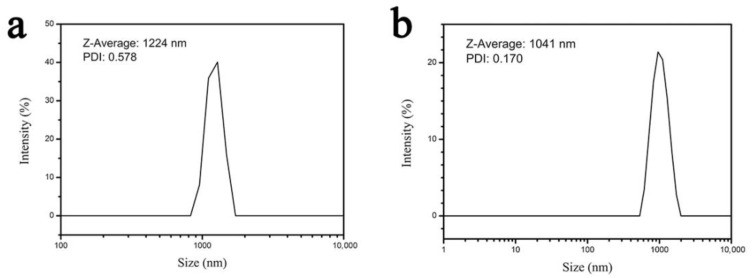
Comparison diragram of particle size (**a**) before and (**b**) after ball milling process.

Figure 3 is a comparison diagram of the particle size of the Mg(OH)_2_ and Al(OH)_3_ mixture samples before and after the ball milling process. Figure 3a is the particle size diagram of the mixture before ball milling, it can be seen that the particle size distribution of the mixture sample is relatively concentrated, with an average particle size of 1224 nm. Figure 3b is the particle size diagram of the mixture after ball milling. It can be seen from the figure that the particle size distribution of the mixture is also relatively concentrated, with an average particle diameter of 1041 nm. It is found that the average particle size of the sample after ball milling process is reduced by 14.95%. During the ball milling process, the particles of the mixture are continuously subjected to intense shearing, friction, impact, and grinding, which reduces the grain size of the mixture sample. The crystals in the mixture undergo plastic deformation, and dislocations multiply and move. Mechanical energy is converted into chemical energy and stored in the crystal defects, which increases the chemical reaction activity of the mixture and greatly reduces the reaction activation energy [28].

**Figure 4:**
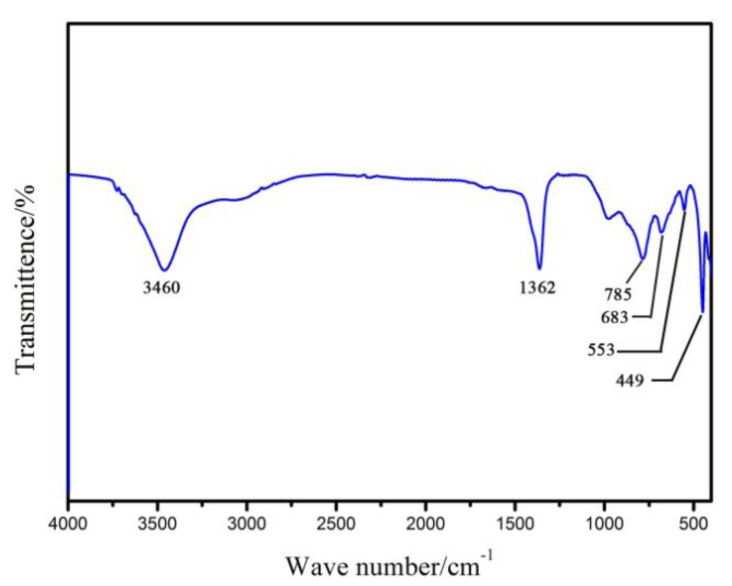
Infrared spectra of Mg_2_Al-CO_3_ LDHs prepared by CO_2_ as carbon sources.

In order to further explore the relevant information of the types of interlayer anions, crystal water and lattice oxygen vibration in the prepared sample, infrared spectroscopy analysis is carried out and the result is shown in Figure 4. It is found that the prepared sample showes a broad absorption peak around 3460 cm^−1^, which is due to the stretching vibration of the hydroxyl attached to the metal ion and the interlayer water molecules. The absorption peaks of the prepared sample at 1362 cm^−1^ is the splitting peaks of the CO asymmetric stretching vibration in the CO_3_^2−^ group. It indicates that the carbonate anion is successfully inserted between the layers. The peaks appearing in the low wave number band represent lattice vibrational vibrations of Mg-O, Al-O, and Al-O-Mg. Among them, the stretching vibration of Al-O bond is near 785 cm^−1^ and 553 cm^−1^ [22].

**Figure 5:**
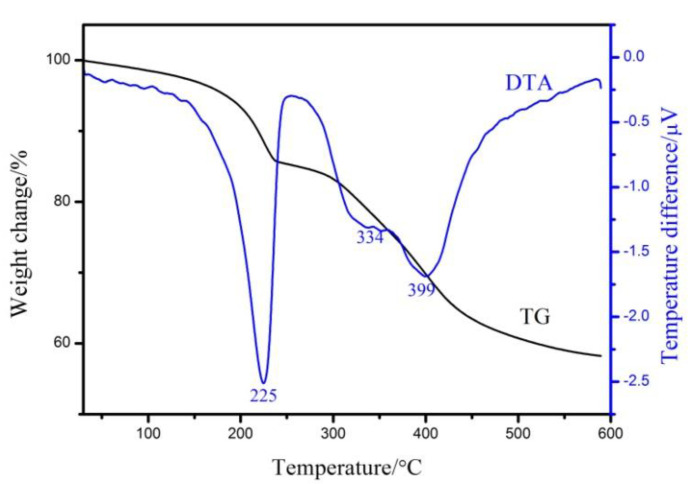
TG-DTA curve of Mg_2_Al-CO_3_ LDHs prepared by basic magnesium carbonate as carbon source.

Figure 5 shows the thermogravimetric-differential thermal analysis (TG-DTA) curve of the prepared sample. It can be seen from the figure that the sample has two obvious stages of mass loss. The first stage (~14%) is between 30 °C and 235 °C, mainly due to the loss of surface absorbed and intercalated water molecules. It can be expressed by the Equation (1).

(1) $${\mathrm{Mg}}_{4}{\mathrm{Al}}_{2}{\left(\mathrm{OH}\right)}_{12}{\mathrm{CO}}_{3}\cdot 3H_{2}O\to {\mathrm{Mg}}_{4}{\mathrm{Al}}_{2}{\left(\mathrm{OH}\right)}_{12}{\mathrm{CO}}_{3}+3H_{2}O$$

The second stage (~28%) is between 235 °C and 450 °C, mainly due to the decompose of hydroxyl in the layer and carbonate ion between the layers [29]. It can be expressed by the Equation (2).

(2) $${\mathrm{Mg}}_{4}{\mathrm{Al}}_{2}{\left(\mathrm{OH}\right)}_{12}{\mathrm{CO}}_{3}\to 3\mathrm{MgO}+{\mathrm{MgAl}}_{2}O_{4}+6H_{2}O+{\mathrm{CO}}_{2}↑$$

The first endothermic peak at 225 °C corresponds to the removal of crystallization water. The second and third peaks at 334 °C and 399 °C corresponds to the dehydroxylation of aluminum hydroxide and magnesium hydroxide, respectively [22].

**Figure 6:**
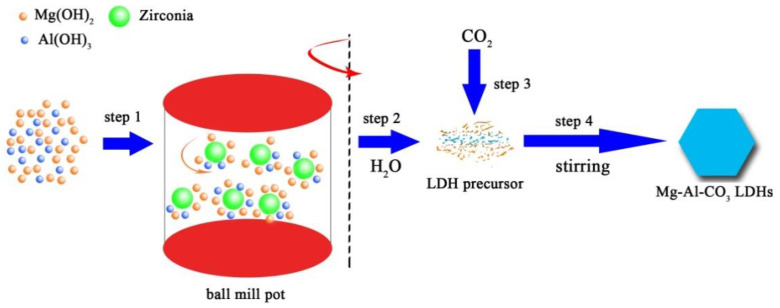
Schematic diagram of mechanochemical preparation method of Mg_2_Al-CO_3_LDHs.

As shown in Figure 6, the weighed mixture is added to a ball mill pot, and the LDH precursor is generated under the action of mechanical force [30,31], which can be combined with carbonate ions in an alkaline solution to form Mg_2_Al-CO_3_ LDHs after stirring 30 h. According to previous studies, the formation mechanism of Mg_2_Al-CO_3_ LDHs prepared by Mg(OH)_2_, Al(OH)_3_ is similar to the formation mechanism of hydrotalcite prepared by co-precipitation. A possible formation mechanism of Mg_2_Al-CO_3_ LDHs prepared by cleaning method occurred according to the following reaction.

(3) $${\mathrm{CO}}_{2}+2{\mathrm{OH}}^{-}\to {\mathrm{CO}}_{3}^{2-}+H_{2}O$$

(4) $$\mathrm{Al}{\left(\mathrm{OH}\right)}_{3}+{\mathrm{OH}}^{-}⇌{\mathrm{AlO}}_{2}^{-}+2H_{2}O⇌\mathrm{Al}{\left(\mathrm{OH}\right)}_{4}^{-}$$

(5) $$\mathrm{Mg}{\left(\mathrm{OH}\right)}_{2}+\mathrm{xAl}{\left(\mathrm{OH}\right)}_{4}^{-}\to {{\mathrm{Mg}}_{1-x}{\mathrm{Al}}_{x}{\left(\mathrm{OH}\right)}_{2}}^{x+}+\mathrm{xMg}{\left(\mathrm{OH}\right)}_{2}\;+2{\mathrm{xOH}}^{-}$$

(6) $${{\mathrm{Mg}}_{1-x}{\mathrm{Al}}_{x}{\left(\mathrm{OH}\right)}_{2}}^{x+}+1/2{\mathrm{xCO}}_{3}^{2-}+{\mathrm{mH}}_{2}O\to {\mathrm{Mg}}_{1-x}{\mathrm{Al}}_{x}{\left(\mathrm{OH}\right)}_{2}{\left({\mathrm{CO}}_{3}\right)}_{1/2x}\cdot {\mathrm{mH}}_{2}O$$

In Equation (3), CO_2_ is absorbed by lye and converted into CO_3_^2−^. In Equation (4), in an alkaline system, Al(OH)_3_ reacts with OH^−^ to produce $\mathrm{Al}{\left(\mathrm{OH}\right)}_{4}^{-}$. In Equation (5), $\mathrm{Al}{\left(\mathrm{OH}\right)}_{4}^{-}$ diffuses into the octahedral voids of OH^−^ accumulation in brucite. Among them, Al^3+^ enters into the octahedral void, substituting a portion of Mg^2+^ in the brucite lattice, forming a coordination structure. This octahedral structure unit forms a network structure through stacking and different connection methods. During the formation of the network structure, defects will inevitably be formed, such as holes and incomplete coordination, resulting in the crystal layer being positively charged. Then, in Equation (6), in order to balance the positive charge carried by the laminate, ${\mathrm{CO}}_{3}^{2-}$ in the solution intercalates into the interlayer through electrostatic interaction, hydrogen bonding and van der Waals force to balance the positive charge on the laminate and gradually forms the hydrotalc structure [32,33].

(7) $$4\mathrm{Mg}{\left(\mathrm{OH}\right)}_{2}+2\mathrm{Al}{\left(\mathrm{OH}\right)}_{3}+{\mathrm{CO}}_{2}+2H_{2}O\to {\mathrm{Mg}}_{4}{\mathrm{Al}}_{2}{\left(\mathrm{OH}\right)}_{12}{\mathrm{CO}}_{3}\cdot 3H_{2}O$$

Equation (7) is the reaction equation, in which the crystal water in hydrotalcite are all from the solution. It can be seen from the Equation (7) that the reactant raw materials are fully utilized.

### 3.2. Thermal Stability of Mg_2_Al-CO_3_ LDHs on PVC

**Figure 7:**
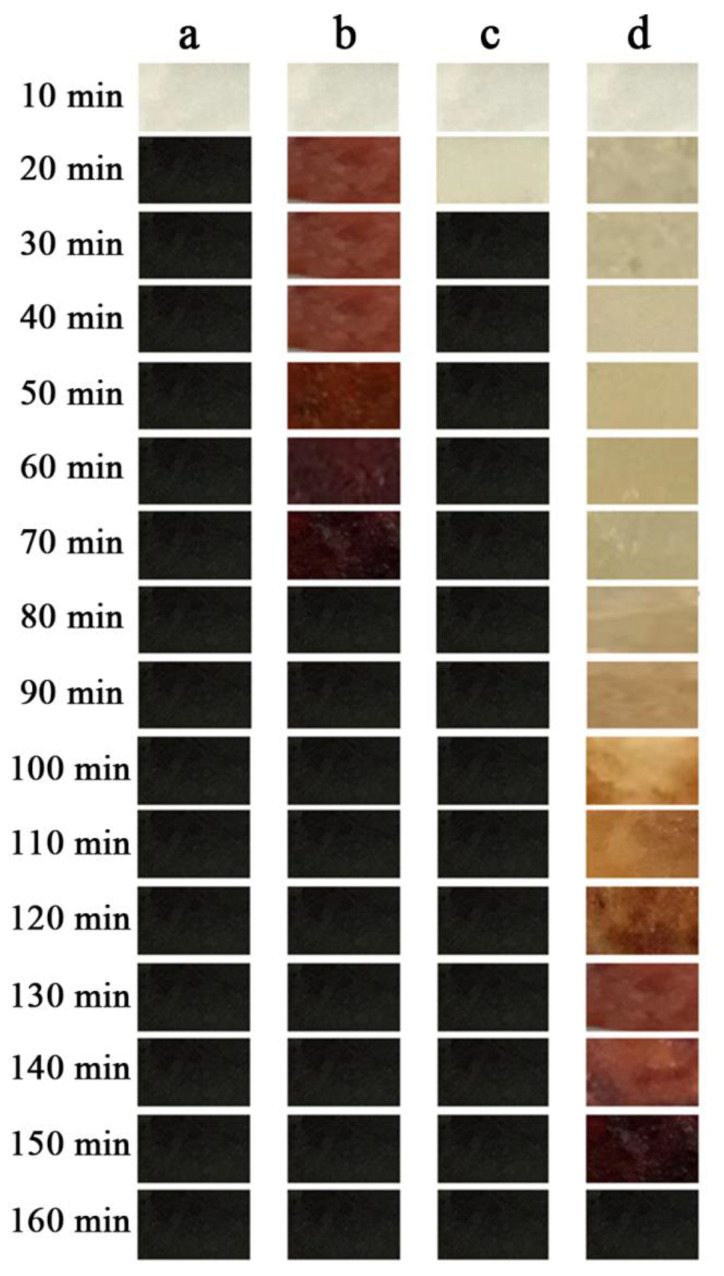
Thermal stability of (**a**) pure PVC and composites of (**b**) PVC + LDHs, (**c**) PVC + ZnSt_2_ and (**d**) PVC + LDHs + ZnSt_2_.

**Figure 8:**
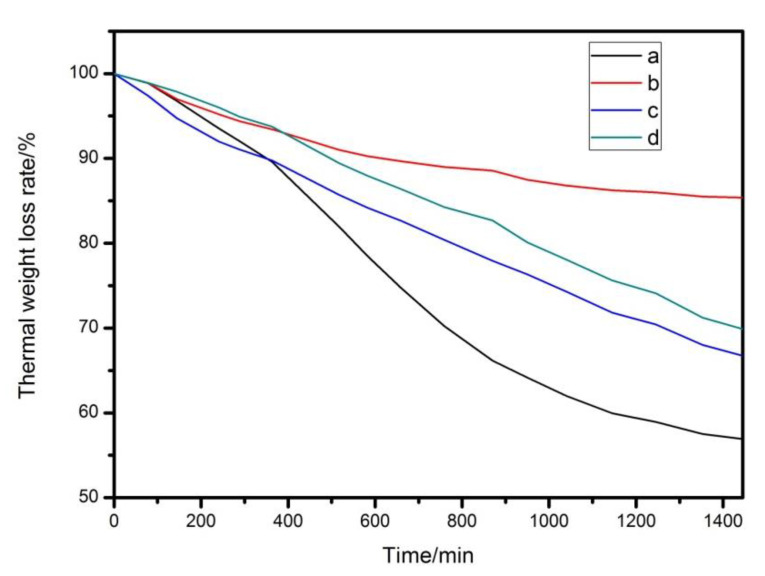
Thermal weight loss rate of (**a**) pure PVC and composites of (**b**) PVC + LDHs, (**c**) PVC + ZnSt_2_ and (**d**) PVC + LDHs + ZnSt_2_.

In order to explore the synergistic thermal stability of Mg_2_Al-CO_3_ LDHs with other stabilizers on PVC, the thermal stability of the samples have been tested. The result of Congo red test of PVC samples are shown in Table 2, and the results of the static oven thermal aging test and thermal weight loss test are shown in Figure 7 and Figure 8. When the Congo red time is longer and the quality loss of PVC in the static oven experiment is less, indicating that the thermal stability of PVC is better.

As shown in Figure 7a and Figure 8, the sample without any addition of heat stabilizer has been completely turned black at 20 min, its thermal stability time is only 8 min according to Congo red test, and it has a weight retention rate of 56.92% at 1440 min under 190 °C condition. Sample with Mg_2_Al-CO_3_ LDHs addition amount of 3 phr begin to color at 20 min and completely turn black at 80 min as shown in Figure 7b. And it has the weight retention rate of 85.37% at 1440 min. Meanwhile, its thermal stability time is 30 min, which is 22 min longer than the sample without any addition of heat stabilizer but it still has a poor initial thermal stability. That is because Mg_2_Al-CO_3_ LDHs belongs to a long-term thermal stabilizer, so it is necessary to add an initial thermal stabilizer into it. Here, zinc stearate (ZnSt_2_) is used as an initial heat stabilizer. As shown in Figure 7c, the sample added with ZnSt_2_ completely turned black at 30 min, has a “zinc burn” phenomenon, its thermal stability time is 12 min. Meanwhile, it has the weight retention rate of 66.74%, which is higher than pure PVC. In Figure 7d, the sample added with LDHs and ZnSt_2_ does not start coloring until 100 min, and has been completely turned black at 160 min. Its thermal stability time is 36 min, which depends on the synergistic effect of LDHs and ZnSt_2_. This is because the fatty acid radicals in zinc stearate can react with allyl chloride on the PVC polymer chain, which can partially eliminate the unstable structure in the long chain of PVC and reduce the initial thermal decomposition rate of PVC. In addition, zinc stearate can absorb HCl gas in the system, and the specific reaction can be expressed by Equations (8) and (9).

(8) $${2\mathrm{RCH}=\mathrm{CHCH}}_{2}{\mathrm{Cl}\;+\;\mathrm{Zn}(C}_{17}H_{35}{\mathrm{COO})}_{2}\to {2R+\mathrm{ZnCl}}_{2}{+2C}_{17}H_{35}{\mathrm{COOCH}}_{2}$$

(9) $${\mathrm{Zn}(C}_{17}H_{35}{\mathrm{COO})}_{2}+2\mathrm{HCl}\;\to {\mathrm{ZnCl}}_{2\;}{+\;2C}_{17}H_{35}\mathrm{COOH}$$

At the same time, it has a weight retention rate of 69.91%, the reason for the decrease in weight retention rate after adding ZnSt_2_ is that the decomposition product ZnCl_2_ is a strong Lewis acid, which will catalyze the degradation of PVC [7].

**Figure 9:**
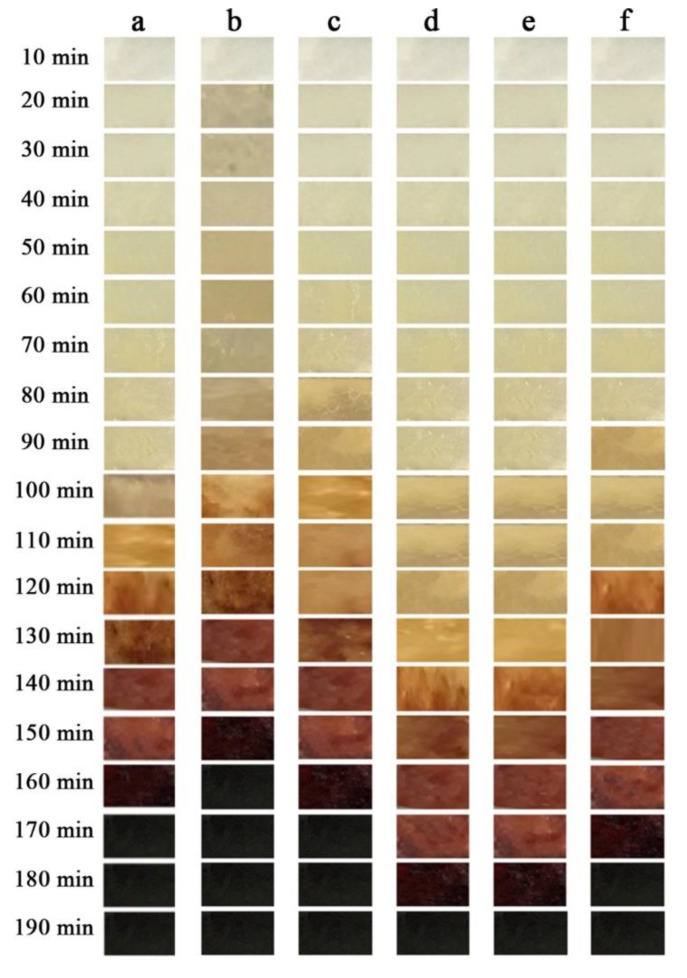
Thermal stability of (**a**) PVC + commercial LDHs + ZnSt_2_, and composite PVC + experimentally prepared LDHs + ZnSt_2_ + (**b**) 0, (**c**) 0.1, (**d**) 0.2, (**e**) 0.3 and (**f**) 0.4 phr Zinc acetylacetonate.

**Figure 10:**
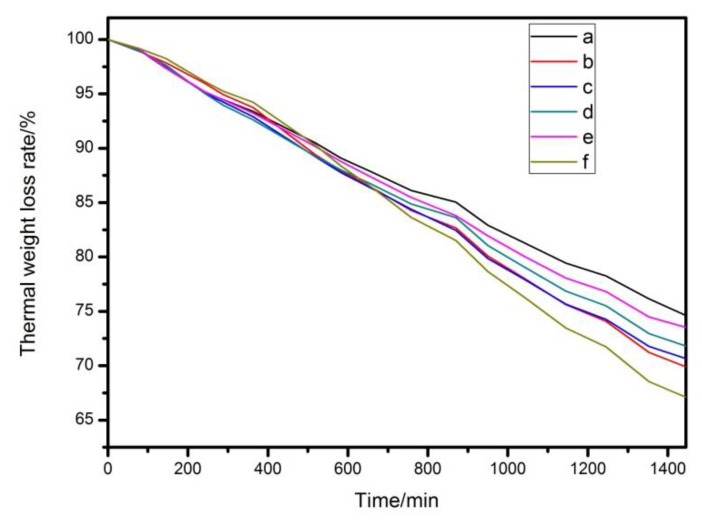
Thermal weight loss rate of (**a**) PVC + commercial LDHs + ZnSt_2_, and composite PVC + experimentally prepared LDHs + ZnSt_2_ + (**b**) 0, (**c**) 0.1, (**d**) 0.2, (**e**) 0.3 and (**f**) 0.4 phr Zinc acetylacetonate.

In order to compare the thermal stability of PVC containing commercial Mg_2_Al-CO_3_ LDHs with the experimentally prepared Mg_2_Al-CO_3_ LDHs, different content of the auxiliary heat stabilizer zinc acetylacetonate compound with experimentally prepared Mg_2_Al-CO_3_ LDHs and zinc stearate, then are added to PVC. The result of Congo red test of PVC samplesare shown in Table 3. The results of the static oven thermal aging test and thermal weight loss test are shown in Figure 9 and Figure 10.

The sample containing commercial Mg_2_Al-CO_3_ LDHs does not start coloring until 110 min, and completely turn black at 170 min, which is 10 min longer than the sample containing experimentally prepared Mg_2_Al-CO_3_ LDHs as shown in Figure 9a,b. The reason is that small amounts of additivesuch as stearic acid is added to commercial Mg_2_Al-CO_3_ LDHs, which can slightly improve its thermal performance. As shown in Figure 9b–f, the samples added with zinc acetylacetonate effectively improved the whiteness in the initial and slightly improved the long-term thermal stability of PVC.

**Figure 11:**
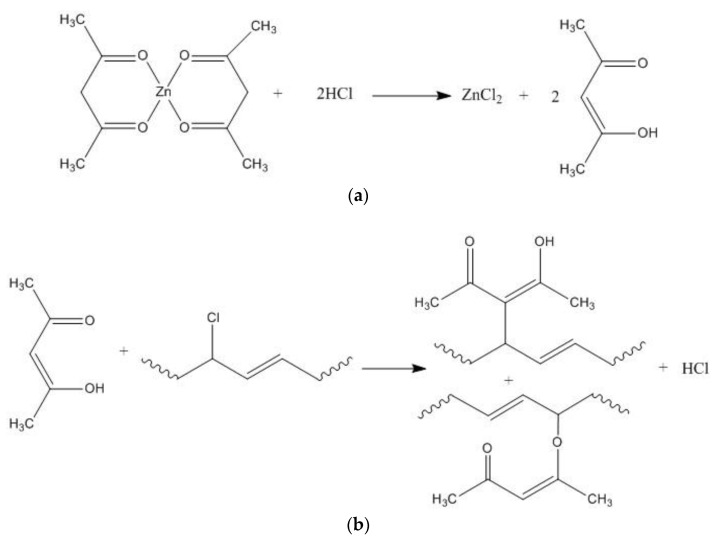
Mechanism of action of zinc acetylacetonate as auxiliary heat stabilizer. (**a**) absorb acid; (**b**) PVC polymer chains crosslinking.

As the content of zinc acetylacetonate increases, the initial and long-term thermal stability of the PVC samples gradually improve, especially the sampled with 0.2 phr zinc acetylacetonate added, which begins to color at 130 min, and completely turn black at 190 min, which is 20 min longer than the sample containing commercial Mg_2_Al-CO_3_ LDHs.Zinc acetylacetonate belongs to β-diketone, which is a kind of auxiliary heat stabilizer. The mechanism of action of zinc acetylacetonate as auxiliary heat stabilizer can be represented in Figure 11. As shown in Figure 11a, zinc acetylacetonate can effectively absorb the HCl gas generated by the thermal degradation of PVC, and the conversion product is acetylacetone. Figure 11b shows that acetylacetone can replace the active allyl Cl atoms on the PVC polymer chain under the catalytic action of ZnCl_2_, reducing the active sites on the PVC polymer chain. Meanwhile, acetylacetone can also be cross-linked with PVC polymer chains to form a stable structure [34].

However, as the content of zinc acetylacetonate continued to increase, the initial and long-term thermal stability of the PVC sample decreased when the addition amount is 0.4 phr as shown in Figure 9f. This is because as the content of zinc acetylacetonate increased, more ZnCl_2_ is produced in the system, which will catalyze the thermal degradation of PVC due to its strong Lewis acidity [7], the initial and long-term thermal stability performance will decrease.

As shown in Table 3 and Figure 10, the thermal stability time of PVC sample containing commercial LDHs is 38 min, which is 3 min longer than experimentally prepared LDHs, and it has a weight retention rate of 74.63% at 1440 min. However, as the content of zinc acetylacetonate increased, the thermal stability time and weight retention rate of PVC sample increases first and then decreases, when 0.3 phr zinc acetylacetonate is added, the PVC sample has a maximum thermal stability time of 46 min, which is 7 min longer than commercial LDHs. Meanwhile, it has weight retention rate of 73.53% at 1440 min. The Congo red test and thermal weight loss test demonstrate the same conclusion as the static oven thermal aging test.

Therefore, when 0.3 phr of zinc acetylacetonate, 2.4 phr Mg_2_Al-CO_3_ LDHs, and 0.6 phr ZnSt_2_ are added to the PVC, the thermal stability of PVC is the best.

### 3.3. Processing Performance Test of Compound Heat Stabilizer Added to PVC

In order to explore the difference in processing performance between the experimentally prepared Mg_2_Al-CO_3_ LDHs and commercial Mg_2_Al-CO_3_ LDHs, a torque rheometer is carried out to test them. The balanceable torque and the mixing process energy were carried out by a Torque Rheometer ZJL-200 (Chang Chun Intelligent Apparatus Co. Ltd., Changchun, China) at 190 °C at a speed of 40 r/min for 10 min.

**Figure 12:**
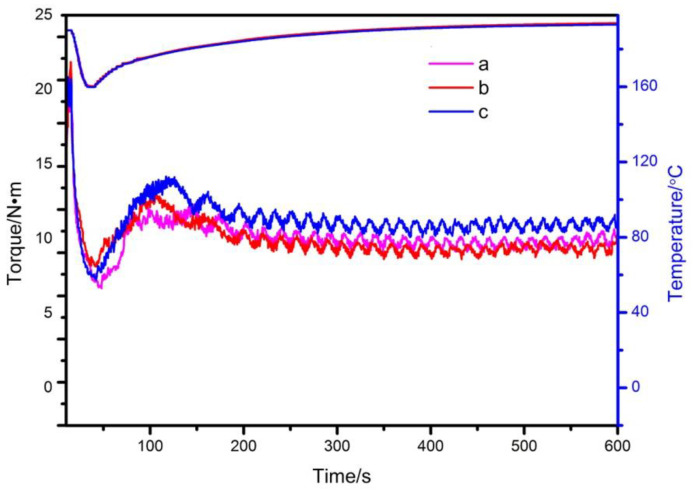
Rheological curvesof composite of PVC + ZnSt_2_ + (**a**) commercial LDHs, or (**b**) experimentally prepared LDHs, or (**c**) experimentally prepared LDHs + Zinc acetylacetonate.

The recipe of the samples are shown in Table 4, the rheological curves are shown in Figure 12 and their rheological data are shown in Table 5.

It can be seen from Figure 12 and Table 5 that the order of maximum torque of the three samples is b > c >a, the balance torque is c > a > b, the melting time is a > c > b, melting temperature is a > c > b, and the balance temperature is c > a > b. The comparison of the three samples show that the maximum torque of sample a is the smallest, which is 20.68 Nm, indicating that its melt viscosity is low, the melt fluidity is the best, which is the easiest to process. Moreover, it has the longest melting time 32 s, and the highest melting temperature 160.3 °C, indicating that sample a is the most difficult to plasticize. Compared with the sample containing commercial Mg_2_Al-CO_3_ LDHs, the maximum torque of the sample containing experimentally prepared Mg_2_Al-CO_3_ LDHs is larger, and the melting time is shorter, indicating that its plasticization is faster, the viscosity of the melt is larger, is harder to process, and the external lubricity of PVC composite is poorer [35]. It is necessary to improve the external lubricity and balance the internal and external lubrication. Both the balance torque and the balance temperature of sample b are low, indicating that PVC compounds consume the least energy during processing. Comparing the sample adding zinc acetylacetonate with the sample without zinc acetylacetonate, the maximum torque is reduced at 21.55 Nm, and the balance torque, balance temperature, the melting time and melting temperature are slightly increased, indicating that the addition of the auxiliary thermal stabilizer zinc acetylacetonate increases the external lubricity of the PVC composite and improves the processing flow properties of the PVC composite. In summary, their processing performance is basically the same, as the processing performance and thermal stability of the prepared samples have reached the commercial level.

## 4. Conclusions

Through mechanochemical methods, the Mg_2_Al-CO_3_ LDHs were successfully prepared by Mg(OH)_2_, Al(OH)_3_, and CO_2_. This method is simple in process, low in cost, high in materials utilization rate, and suitable for large-scale production.

The samples Mg_2_Al-CO_3_ LDHs were used as a heat stabilizer in PVC. The result shows that when 2.4 phr Mg_2_Al-CO_3_ LDHs, 0.3 phr ZnSt_2_ and 0.3 phr of zinc acetylacetonate are added to the PVC, the thermal stability time of PVC can reach 190 min under 180 °C oven test conditions, which is 20 min longer than the PVC composite containing 2.4 phr commercial Mg_2_Al-CO_3_ LDHs and 0.6 phr ZnSt_2_. Meanwhile, the result of Congo red test shows that its thermal stability time is 46 min, which is 7 min longer than the time of commercial Mg_2_Al-CO_3_ LDHs. Moreover, its processing performance is basically the same as the PVC containing commercial Mg_2_Al-CO_3_ LDHs.

## Figures and Tables

**Table 1:** Crystal parameters of LDHs.

Sample	d_003_/Å	d_006_/Å	d_222_/Å	d_225_/Å	d_228_/Å	d_600_/Å	d_603_/Å
Standard card	7.570	3.778	2.570	2.281	1.932	1.524	1.493
a	7.586	3.789	2.576	2.286	1.938	1.524	1.493

**Table 2:** The result of Congo red test of PVC samples.

Label	PVC/g	LDHs/phr	ZnSt_2_/phr	Thermal Stability Time of PVC Samples (min)
a	100	0	0	8
b	100	3.0	0	30
c	100	0	0.6	12
d	100	2.4	0.6	36

**Table 3:** The result of Congo red test of zinc acetylacetonate added to the PVC samples.

Label	LDHs Category	PVC/g	LDHs/phr	ZnSt_2_/phr	Zinc Acetylacetonate/phr	Thermal Stability Time of PVC Samples (min)
a	commercial	100	2.4	0.6	0	39
b	experimental	100	2.4	0.6	0	36
c	experimental	100	2.4	0.5	0.1	38
d	experimental	100	2.4	0.4	0.2	45
e	experimental	100	2.4	0.3	0.3	46
f	experimental	100	2.4	0.2	0.4	41

**Table 4:** Rheology test recipe of the samples.

Label	PVC/g	DOP/g	CaCO_3_/g	Stearic Acid/g	LDHs Category	LDHs/phr	ZnSt_2_/phr	zinc Acetylacetonate/phr
a	100	10	30	0.5	commercial	2.4	0.6	0
b	100	10	30	0.5	experimental	2.4	0.6	0
c	100	10	30	0.5	experimental	2.4	0.3	0.3

**Table 5:** Rheological data of the samples.

Label	Maximum Torque/(Nm)	Balance Torque/(Nm)	Melting Time/s	Melting Temperature/°C	Balance Temperature/°C
A	20.68	9.73	32	160.3	193.0
B	22.09	9.35	26	159.8	192.7
C	21.55	10.91	27	160.1	193.5

## References

[1] Yang H., Yang Z.H. (2018). The effect of sodium stearate-modified hydrocalumite on the thermal stability of poly(vinyl chloride). J. Appl. Polym. Sci..

[2] Korkusuz Ç., Demir A.P.T. (2019). Evaluation of the thermal stabilization behavior of hydrotalcite against organic stabilizers for plasticized PVC films. Polym. Bull..

[3] Mohamed N.A., Sabaa M.W., Oraby E.H., Yassin A.A. (2003). Organic thermal stabilizers for rigid poly(vinyl chloride) VII. Effect of mixing 2-benzimidazolyl-omega-phenylpropenylidineacetonitrile with some commercial stabilizers. Polym. Degrad. Stab..

[4] Xu X., Chen S., Tang W., Qu Y., Wang X. (2013). Synthesis and application of uracil derivatives as novel thermal stabilizers for rigid poly(vinyl chloride). Polym. Degrad. Stab..

[5] Shu W.Y., Liu Y.N., Chen Q.Y. (2002). Synthesis of antimony tris(mercaptoethyl carboxylates) as thermal stabilizer for polyvinyl chloride. Trans. Nonferrous Metal Soc. China.

[6] Gupta S., Agarwal D., Banerjee S. (2009). Thermal stabilization of poly(vinyl chloride) by hydrotalcites, zeolites, and conventional stabilizers. J. Vinyl Addit. Technol..

[7] Xu X., Chen S., Tang W., Qu Y., Wang X. (2014). Investigation of basic zinc cyanurate as a novel thermal stabilizer for poly(vinyl chloride) and its synergistic effect with calcium stearate. Polym. Degrad. Stab..

[8] Wang M., Xu J., Wu H., Guo S. (2006). Effect of pentaerythritol and organic tin with calcium/zinc stearates on the stabilization of poly(vinyl chloride). Polym. Degrad. Stab..

[9] Liu L., Cheng M., Yang Z. (2018). Improved performance of flower-like ZnAl LDH growing on carbon nanotubes used in zinc–nickel secondary battery. Electrochim. Acta.

[10] Wang G., Yang M., Li Z., Lin K., Jin Q., Xing C., Hu Z., Wang D. (2013). Synthesis and characterization of Zn-doped MgAl-layered double hydroxide nanoparticles as PVC heat stabilizer. J. Nanopart. Res..

[11] Halajnia A., Oustan S., Najafi N., Khataee A., Lakzian A. (2013). Adsorption–desorption characteristics of nitrate, phosphate and sulfate on Mg–Al layered double hydroxide. Appl. Clay Sci..

[12] Takahashi N., Hata H., Kuroda K. (2010). Anion Exchangeable Layered Silicates Modified with Ionic Liquids on the Interlayer Surface. Chem. Mater..

[13] Meloni D., Monaci R., Solinas V., Auroux A., Dumitriu E. (2008). Characterisation of the active sites in mixed oxides derived from LDH precursors by physico-chemical and catalytic techniques. Appl. Catal. A Gen..

[14] Huang J., Yang Z.-H., Wang R., Zhang Z., Feng Z., Xie X. (2015). Zn–Al layered double oxides as high-performance anode materials for zinc-based secondary battery. J. Mater. Chem. A.

[15] Jiang Y., Yang Z., Liu L., Meng J., Cui F. (2019). Zn-Al layered double hydroxide growing on the substrate of graphene and polypyrrole composite as anode material for Zn-Ni secondary battery. Mater. Lett..

[16] Meng J., Yang Z., Liu L., Cui F., Jiang Y. (2019). The in-situ growth of zinc-aluminum hydrotalcite on hollow carbon spheres and its application as anode material with long cycle life for zinc-nickel secondary battery. J. Alloys Compd..

[17] Hu X., Zhu X., Sun Z. (2019). Efficient flame-retardant and smoke-suppression properties of MgAlCO3-LDHs on the intumescent fire retardant coating for steel structures. Prog. Org. Coat..

[18] Hu X., Zhu X., Sun Z. (2020). Fireproof performance of the intumescent fire retardant coatings with layered double hydroxides additives. Constr. Build. Mater..

[19] Liu Y., Yang Z., Xie X., Huang J., Wen X. (2015). Layered Double Oxides Nano-flakes Derived from Layered Double Hydroxides: Preparation, Properties and Application in Zinc/Nickel Secondary Batteries. Electrochim. Acta.

[20] Yan J., Yang Z. (2017). Intercalated hydrotalcite-like materials and their application as thermal stabilizers in poly(vinyl chloride). J. Appl. Polym. Sci..

[21] Yi S., Yang Z., Wang S.-W., Liu D.-R., Wang S.-Q., Liu Q.-Y., Chi W.-W. (2011). Effects of MgAlCe-CO3 layered double hydroxides on the thermal stability of PVC resin. J. Appl. Polym. Sci..

[22] Lin Y.-J., Li D.-Q., Evans D.G., Duan X. (2005). Modulating effect of Mg–Al–CO3 layered double hydroxides on the thermal stability of PVC resin. Polym. Degrad. Stab..

[23] Zhang X., Zhao T., Pi H., Guo S. (2011). Preparation of intercalated Mg-Al layered double hydroxides and its application in PVC thermal stability. J. Appl. Polym. Sci..

[24] Zhao X., Cao J.-P., Zhao J., Hu G.-H., Dang Z.-M. (2014). A hybrid Mg–Al layered double hydroxide/graphene nanostructure obtained via hydrothermal synthesis. Chem. Phys. Lett..

[25] Gupta S., Agarwal D.D., Banerjee S. (2012). Role of Hydrotalcites Cations in Thermal Stabilization of Poly (Vinyl Chloride). Int. J. Polym. Mater..

[26] Zhao Y., Li F., Zhang R., Evans A.D.G., Duan X. (2002). Preparation of Layered Double-Hydroxide Nanomaterials with a Uniform Crystallite Size Using a New Method Involving Separate Nucleation and Aging Steps. Chem. Mater..

[27] Zhao T.-T., Feng G.-H., Chen W., Song Y.-F., Dong X., Li G.-H., Zhang H.-J., Wei W. (2019). Artificial bioconversion of carbon dioxide. Chin. J. Catal..

[28] Bao T.N., Tegus O., Hasichaolu, Ning J., Narengerile (2018). Preparation of Black Phosphorus by the Mechanical Ball Milling Method and its Characterization. Solid State Phenom..

[29] Zhitova E.S., Greenwell H.C., Krzhizhanovskaya M.G., Apperley D.C., Pekov I.V., Yakovenchuk V.N. (2020). Thermal Evolution of Natural Layered Double Hydroxides: Insight from Quintinite, Hydrotalcite, Stichtite, and Iowaite as Reference Samples for CO_3_- and Cl-Members of the HydrotalciteSupergroup. Minerals.

[30] Wang B., Qu J., Li X., He X., Zhang Q. (2016). Precursor Preparation to Promote the Adsorption of Mg-Al Layered Double Hydroxide. J. Am. Ceram. Soc..

[31] Li Z., Chen M., Ai Z., Wu L., Zhang Q. (2018). Mechanochemical synthesis of CdS/MgAl LDH-precursor as improved visible-light driven photocatalyst for organic dye. Appl. Clay Sci..

[32] Eliseev A., Lukashin A.V., Vertegel A.A., Tarasov V.P., Tret’Yakov Y.D. (2002). A Study of Crystallization of Mg–Al Double Hydroxides. Dokl. Chem..

[33] Yang Y., Zhao X., Zhu Y., Zhang F. (2011). Transformation Mechanism of Magnesium and Aluminum Precursor Solution into Crystallites of Layered Double Hydroxide. Chem. Mater..

[34] Belhanechebensemra N., Van H.T., Guyot A., Gay M., Carette L. (1989). Thermal dehydrochlorination and stabilization of poly(vinylchloride) in solution: Part IV—Synergistic effects of β-diketone compounds and metal soap stabilizers. Polym. Degrad. Stab..

[35] Shnawa H.A. (2020). Characterization of processing, rheological and dynamic mechanical thermal properties of PVC stabilized with polyphenol-based thermal stabilizer. J. Therm. Anal. Calorim..

